# Assessing Cognitive Estimation and Its Effects on Community Integration in People with Acquired Brain Injury Undergoing Rehabilitation

**DOI:** 10.1155/2017/2874819

**Published:** 2017-07-26

**Authors:** Dónal G. Fortune, Helen L. Richards

**Affiliations:** ^1^Department of Psychology, University of Limerick, Limerick, Ireland; ^2^Department of Psychology, Mercy University Hospital, Cork, Ireland

## Abstract

The purpose of the present study was to examine the convergent and divergent validity of the Biber Cognitive Estimation Test (BCET) in individuals with ABI undergoing postacute rehabilitation and to assess the measure's ability to account for unique variance in community integration following rehabilitation. Participants with ABI referred for postacute rehabilitation (*N* = 201) were assessed on the BCET and a number of other neuropsychological tests that have been demonstrated to rely on aspects of executive processing (Trail-Making Test, Modified Six Elements Test, and verbal fluency measures) and the Repeatable Battery for the Assessment of Neuropsychological Status (RBANS). Internal consistency of the total BCET was good; however, interpretable solutions for existing subscales were not discerned. The BCET total score demonstrated positive associations with tests of executive functioning; however, it was also significantly associated with more general aspects of neuropsychological functioning suggesting that it does not solely assess executive processes in ABI patients undergoing rehabilitation. Hierarchical multiple regression suggested that the BCET accounted for significant additional variance in community integration after severity of disability, executive functioning, and more general aspects of neuropsychological status were statistically controlled. While the subscale structure of the BCET may be somewhat inconsistent, the total scale score accounts for some unique variance in pragmatic rehabilitation outcome and may be a useful tool in postacute rehabilitation assessment protocols.

## 1. Introduction

The ability to provide reasonable numerical estimates from available but incomplete information is an ability not normally acquired directly through formal education, yet it is an important aspect of problem solving that is regularly called upon in everyday life. Previously acquired information may not always have direct relevance to a particular estimation problem and, as such, problems in providing reasonable or plausible cognitive estimations are related not simply to an abstract problem in processing numbers, but also to probabilistic judgements (mapping components) arising from domain-specific reasoning and general heuristics [1, 2]. Failure in such estimation abilities is likely to have outcomes that may significantly impact a person's everyday life [3, 4]: for example, incorrectly estimating whether one might have time to visit the post office before one's bus is due to leave or erroneously estimating how much food to prepare for a particular number of people and so on. According to MacPherson and colleagues [5], “to produce reasonable cognitive estimates, individuals need to identify and select the appropriate cognitive set, retrieve and manipulate particular details or estimates from that cognitive set, monitor the appropriateness of their response and repeat the procedure if necessary to produce a better estimate” (p.1). Therefore, cognitive estimation is a complex and strategic cognitive process of creating an approximation based on information that is available but incomplete [4]. Given this cognitive complexity, estimation abilities are likely to have significant impacts for people with a range of neurological diagnoses, especially acquired brain injury, and may also impact on the outcome of rehabilitation endeavour in this patient group. This necessitates formal examination of the accuracy of measurement of the construct as well as examination as to whether a relationship exists between estimation abilities and the ultimate goal of brain injury rehabilitation, the reintegration of the person into their community.

The first formal examination and detailed assessment of estimation abilities were performed on individuals with lesions to the frontal lobes [4]. This landmark study described a patient who, despite having preserved general cognitive functioning, demonstrated significant and wide ranging difficulties in his ability to provide reasonable cognitive estimates. For example, he estimated the height of the highest building in London as between 18,000 and 20,000 feet and that the largest fish in the world was a trout which was over 3 metres long. Estimation difficulties are not solely limited to individuals with such discrete frontal lobe injury, and problems in estimation abilities have been reported in adults with Alzheimer's disease [6–9], viral encephalitis [10], frontotemporal dementia [9, 11], Huntington's disease [12], Korsakoff's syndrome [13, 14], Parkinson's disease [15–17] (however, see Scarpina et al. [18] for a recent finding of no difference between PD patients and controls), vascular dementia [19, 20], traumatic brain injury [21–23], major depressive disorder [24], and schizophrenia [25–27]. This breadth of effect across a broad number of conditions would suggest that investigation of estimation abilities in terms of measurement and possible effects on outcome is worthy of investigation. Moreover, the finding of estimation difficulties in these relatively disparate patient groups may suggest that estimation abilities may not entirely depend solely on executive processes. Indeed, Taylor and O'Carroll [28] reported that patients in their sample (with a wide range of neurological diagnoses) did not significantly differ in terms of their estimation abilities as a result of whether their injuries were the result of anterior or posterior injury. Similarly, in people participating in a formal neurorehabilitation programme, estimation abilities were correlated with a number of cognitive domains, including executive as well as nonexecutive performance domains [23]. Moreover, Silverberg et al. [22] found that assessments of cognitive estimation showed a stronger correlation with nonspecific tests than with executive functioning measures and did not predict functional status as measured by the disability rating scale. Taken together, these findings suggest that estimation abilities may be compromised by a range of neurological disorders and be associated with impairments in a number of functional performance domains. It would be reasonable to hypothesise that estimation abilities may in turn affect longer term outcomes for patients with brain injury and their eventual integration in their community.

Community integration of people with ABI rests on the premise that such individuals should have the same rights and be involved in community life like any other member of their community. It is generally considered to represent a person's ability to carry out everyday activities in their home and community, enjoy interaction with its members, and participate in some aspect of productivity in the community. While community integration is also the ultimate goal of rehabilitation following ABI, formal examination of the construct and its possible relationship to variation in community integration outcomes in the context of rehabilitation have not been undertaken to date. This mandates a comprehensive examination of a widely used measure of cognitive estimation and its influence on more distal community integration outcomes.

There is no clear consensus on the use of a particular measure of cognitive estimation that has been rigorously developed and standardised. In terms of measurement of the construct, it has been observed [21] that there are almost as many versions of the cognitive estimation task as there are research studies on cognitive estimation with most researchers developing their own estimation measure, often changing both the questions and scoring method. A number of estimation tests have been developed either as new formal instruments and amendments to published tests or as translations to be used in particular language populations [5, 8, 29–34]. Therefore, in the current study, the Biber Cognitive Estimation Test (BCET) [29] was utilised as an assessment of cognitive estimation as it has good norms, absence of specified units for response, an impairment cut-off score, equal number of questions from a number of practical estimation domains (proposed subscales addressing estimations of weight, time/age, quantity, and distance/length), and has been used previously in a rehabilitation population [22].

The aims of the study were as follows: firstly, to examine the latent variable structure of the BCET in relation to the subscale assessment domains of quantity, time/age, length, and weight in a sample of people referred for postacute rehabilitation following acquired brain injury; secondly, given the question posed by previous research as to whether estimation is associated more with performance on executive or more general cognitive function assessments, to investigate the convergent and divergent validity of cognitive estimation using established neuropsychological tests that are considered to represent executive and more general measures of cognitive function; thirdly, to assess what aspects of cognitive function measured by the battery of neuropsychological tests may be responded to differently in an impaired estimation group relative to an unimpaired estimation group; and finally, to investigate whether the BCET adds any unique variance to prediction of community integration, given that community integration is the ultimate goal of rehabilitation, and impairments in estimation ability may have real-world consequences in terms of such community integration aims (the extent to which people live, participate, and socialize in their own community).

## 2. Method

### 2.1. Participants

A total of 201 participants who had sustained an acquired brain injury and who were referred for postacute neurorehabilitation services in the South and Mid-West of Ireland participated in the study. All participants engaged in a programme of accredited and individualized Home and Community Based Rehabilitation (HCBR). HCBR is defined by the Commission of Accreditation of Rehabilitation Facilities (CARF), as a programme of postacute rehabilitation that provides integrated, case managed, and outcome focused rehabilitation. In the HCBR model, services are developed from a comprehensive needs assessment and focus on the expectations and outcomes identified in treatment planning with the person served by the programme. The positive effects of such individualized community based programmes are well established [35].

Inclusion criteria were participants accessing ABI-specific neurorehabilitation services (home and community rehabilitation), confirmed medical diagnosis of ABI, age over 18 years and not older than 65 years at study end, and English as a first language. Exclusion criteria were the presence of aphasia/communication, comprehension, or significant visual difficulty that necessitated an alternative assessment protocol.

The principal purpose of recruiting ABI rather than a single cause of brain injury (i.e., traumatic brain injury alone or stroke alone) was that it would reflect the possible ecological or real-world rehabilitation usage of the measure of cognitive estimation and permit an examination of its ability to predict the ultimate goal of rehabilitation (community integration) over and above the standard assessments undertaken in this group.

All participants gave informed consent. Participants were predominantly male (men *n* = 150; 74.6%; women *n* = 51; 25.4%) and were aged between 19 and 63 years (mean 42.18, SD = 11.64). Age at onset of brain injury ranged from 1 to 60 years (mean 33.43, SD 14.48), and duration of injury ranged from 2 years to 20 years (mean 8.44, SD 5.14). Twenty percent of participants (*n* = 40) completed more than 14 years of education, 33% (*n* = 67) 12 to 14 years, and 38% (*n* = 76) 8 to 12 years, and 9% of participants (*n* = 18) completed less than 8 years of formal education. Participants' functional disability was assessed on the Mayo Portland Adaptability Index [36] during their programme. A range of standardised neuropsychological measures (Biber Cognitive Estimation Test [29], Repeatable Battery for the Assessment of Neuropsychological Status [37], verbal fluency measures [38], Modified Six Elements Test [39], and Trail-Making Test [40]) were used to measure an array of current cognitive functioning at induction to the study. Participants were subsequently assessed on the Community Integration Questionnaire [41] in their home community on completion of their active rehabilitation programme.

### 2.2. Cause of Injury

49.5% of participants sustained a TBI (*n* = 99), 35% had stroke (*n* = 71), and 15.5% (*n* = 31) had other diagnoses including neoplasm (7%, *n* = 14), anoxia (3.5%, *n* = 7), encephalitis (3%, *n* = 6), and aboulia (2%, *n* = 4). Due to the small number of participants in some subcategories of brain injury, 3 categories were created for subsequent analysis: traumatic brain injury, stroke, and a third category comprising neoplasm, anoxia, aboulia, and encephalitis.

### 2.3. Functional Severity of Injury

Participants were assessed on the Mayo Portland Adaptability Index 4 [36], a widely used assessment of ABI-induced disability in rehabilitation contexts. It provides assessment of functioning in terms of a person's abilities (e.g., mobility, cognitive functioning, and communication), adjustment (e.g., pain, mood, and fatigue), and participation (e.g., social contact, independent living, and employment). The MPAI-4 was thus used as a sensitive index of severity of brain injury-induced disability resulting from a variety of causes given the absence of formal severity assessments at time of injury and the heterogeneous nature of the acquired brain injury sample.

Mean MPAI *T* score for the sample was 39.03 (SD = 12.52). *T*-scores were used as an assessment of the severity of functional limitations such that scores less than 50 indicated mild to moderate limitations, while scores above 60 were suggestive of more severe limitations arising from ABI. One hundred and four participants (51.7%) had disability in the moderate to severe range, while 97 participants (48.3%) had disability scores in the mild to moderate category.

### 2.4. Procedure

The study was approved by the local research ethics committee (ABI Ireland LREC, 44 Northumberland Road, Dublin, Ireland, number ABII 1017) and all participants provided informed consent commensurate with the declaration of Helsinki. The study adhered to standardised reporting guidelines for cohort studies (STROBE). All participants completed the following assessment measures.

### 2.5. Measures

#### 2.5.1. Biber Cognitive Estimation Test (BCET)

The BCET [29] is a 20-item clinical interview that sets out to assess a person's ability to provide reasonable estimates of weight, distance/length, time/age, and quantity. The responses to the BCET cannot be answered by relying solely on a persons' fund of knowledge and require novel reasoning in order to prove a reasonable estimate. The development of the BCET sought to provide an easily administered and objectively normed instrument of cognitive estimation, with a broadly defined normal range for each item. Individual items are scored correct or incorrect with a range of scores of 0 to 20. Scores between the 5th and 95th percentile of a normative sample are taken as representing a correct score, with scores beyond this range being incorrect. There is a published cut-off score of 15.6, where scores below this threshold may be suggestive of clinically relevant problems in estimation abilities. The originator of the BCET also suggests that 4 subscales underlie the scale with questions targeting the constructs of (1) distance/length, (2) quantity, (3) time/age, and (4) weight.

#### 2.5.2. Repeatable Battery for Assessment of Neuropsychological Status (RBANS)

The RBANS [37] is a brief cognitive screening battery consisting of 12 subtests which are used to create index scores in the following five cognitive domains: immediate memory, visuospatial/constructional skills, language, attention, and delayed memory. A total score is created by summing the five index scores. A total score of 100 is considered average with a standard deviation (SD) of 15. Research has supported the clinical application of the RBANS as a neuropsychological tool within people with ABI [42, 43].

#### 2.5.3. Phonemic and Semantic Verbal Fluency

Participants were required to generate as many words as possible that either begin with a particular letter, for example, F, A, and S (phonemic fluency), or that belong to a specific semantic category, fruits, vegetables, and animals (semantic fluency) within a specified time interval. These measures are commonly used to assess the organisation and integrity of lexical and semantic memory representations and executive functions. Phonemic and semantic verbal fluency relies on a partially different network of brain regions [44], and there is some evidence that frontal lobe damage may be more likely to result in impairment to phonemic fluency, whereas temporal lobe damage may preferentially impair semantic rather than phonemic verbal fluency [45].

#### 2.5.4. Modified Six Elements Test

The Modified Six Elements Test (MSET) [39] consists of three tasks (simple arithmetic, written picture naming, and dictation), each of which consists of two parts (subtasks A and B). Participants were informed that they have 10 mins to work on at least a part of each of these six subtasks and are also not permitted to switch between two parts (subtasks) of the same task. This rule requires participants to engage in task switching throughout the test. The MSET has been used in a range of patient groups including persons with brain injury [46–48]. The MSET has been demonstrated to be a very sensitive test of executive problems [46] and to be a valid discriminator between people with anterior and posterior brain injury [49].

#### 2.5.5. Trail-Making Test (TMT)

The TMT [40] was used to measure cognitive and motor speed and mental flexibility and consists of two parts, A and B. In Part A, the participant is instructed to connect numbers in order, beginning with 1 and ending with 25, as quickly as possible. In Part B, the participant is instructed to connect numbers and letters in an alternating pattern (1-A-2-B-3-C, etc.) as quickly as possible. There are a number of derived scores for the TMT and in the current study the B − A difference score and the B/A ratio score were used. The B − A difference score was utilised as it is suggested to provides a purer indicator of executive control processes, by minimizing visuoperceptual and motor demands [50]. The B/A ratio score was also utilised as it is suggested to reduce the influence of psychomotor demands and controls for intrasubject variability factors [51].

#### 2.5.6. Community Integration Questionnaire (CIQ)

Community Integration of participants was assessed by use of the Community Integration Questionnaire (CIQ) [41]. The CIQ comprises 15 items assessing effective role performance in three domains: Home integration (active participation in the operation of the home or household), Social integration (participation in social activities outside the home) and Productivity (regular performance of work, school or volunteer activities). Internal consistency in previous studies has been reported as good, with Cronbach's alpha's ranging from 0.76–0.84 for total scale scores [41, 52]. The CIQ is predominately linked to the major life areas (35%), community, social and civic life (31%) and domestic life (19%) chapters of the WHO ICF [53].

### 2.6. Data Analysis

Q-Q plots and Shapiro-Wilk's test were used to examine the distribution of data. Because of the nonnormal distribution of trail-making scores in both raw (TMT A, TMT B) and derived measures (TMT B/A, TMT B − A) and in the Modified Six Elements Test (MSET), transformation was applied before further analysis to render this data suitable for parametric analysis. Observed data showed no significant deviation from normality (Shapiro-Wilk's statistic < 0.981, *p*'s > 0.11). Fourteen cases had some missing data from their assessments. No participant had missing data in excess of 7% on the assessment measures, and no participant had missing data on the neuropsychological assessments. To assess whether the data was Missing Completely at Random (MCAR) or whether there was an underlying pattern to this missing data, Little's MCAR test was utilised. MCAR values ranged from *χ*^2^ = 6.399, *p* = 0.93, to *χ*^2^ = 78.016, *p* = 0.97, which suggested that the small amount of missing data was randomly missing. Therefore, these cases were retained and the standard expectation-maximisation procedure was utilised to maximise power. Means and standard deviations were calculated for the main measures and subscales as appropriate. Analysis of Variance with Scheffe post hoc test was used to examine the effects of educational attainment and cause of ABI on BCET scores. Independent samples *t*-test was used to examine mean differences between scores on the neuropsychological measures and disability total score as a function of BCET cut-off scores, and Chi Square analysis examined the association between the BCET cut-off and levels of disability severity. Relationships between cognitive estimation and demographic variables, neuropsychological measures, disability, and community integration were undertaken by Pearson correlation coefficient. Latent variable structure of the BCET was undertaken via the Categorical Principal Components Analysis (CatPCA) program. Logistic regression analysis was used to examine neuropsychological factors associated with cognitive estimation scores that were below threshold, and hierarchical multiple regression was used to explore whether the BCET accounted for unique variance in community integration. All data were analysed using SPSS 24.

## 3. Results

Descriptive statistics (means and standard deviations) for the BCET, TMT, verbal fluency, MSET, and the six indices of the RBANS are presented in Table 1. Internal consistency of the BCET total scale score was good (*α* = 0.86), with subscale scores being acceptable for estimates of distance/length (*α* = 0.73) and weight (*α* = 0.69) and somewhat lower for quantity (*α* = 0.57) and time/age (*α* = 0.62). Correlations between each of the four domains and the total scale score were as follows: quantity (*r* = 0.62), weight (*r* = 0.60), time/age (*r* = 0.55), and distance/length (*r* = 0.71).

### 3.1. Latent Variable Structure of BCET

In order to appropriately examine the latent variable structure of the BCET, categorical PCA (CatPCA) was utilised. CatPCA is a relatively new algorithmic model and can be considered the nonlinear equivalent of PCA. It is especially suitable for the dimension reduction problem with categorical variables, as it accounts for the nature of items, the different role of items in determining the measure, and the possible multidimensionality of the underlying concept [54, 55]. The quantitative measures additionally have coordinates that allow the categories or dimensions to be represented in a geometric display, thus making data interpretation more straightforward.

A scree plot was firstly examined to determine the most parsimonious number of components to retain in the analysis. Nonlinear PCA solutions are not nested, so the position of the scree plot “elbow” can move with the number of components. In the present analysis, different dimensionalities consistently placed the elbow at the fourth component.

**Figure 1:**
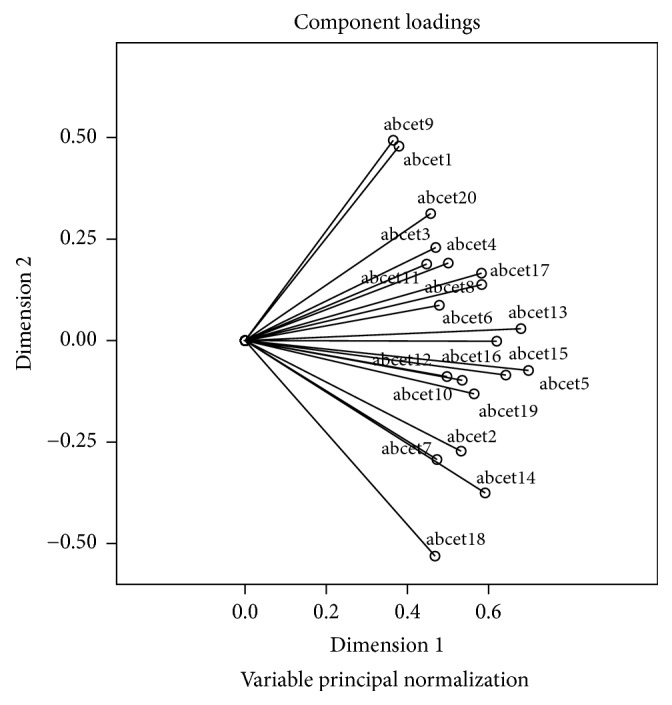
CatPCA biplot of dimensions 1 and 2.

**Figure 2:**
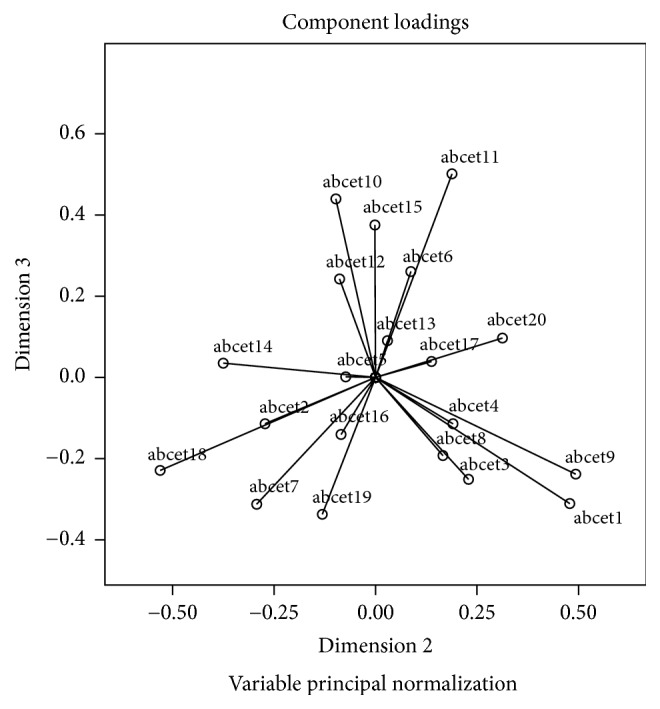
CatPCA biplot of dimensions 2 and 3.

**Figure 3:**
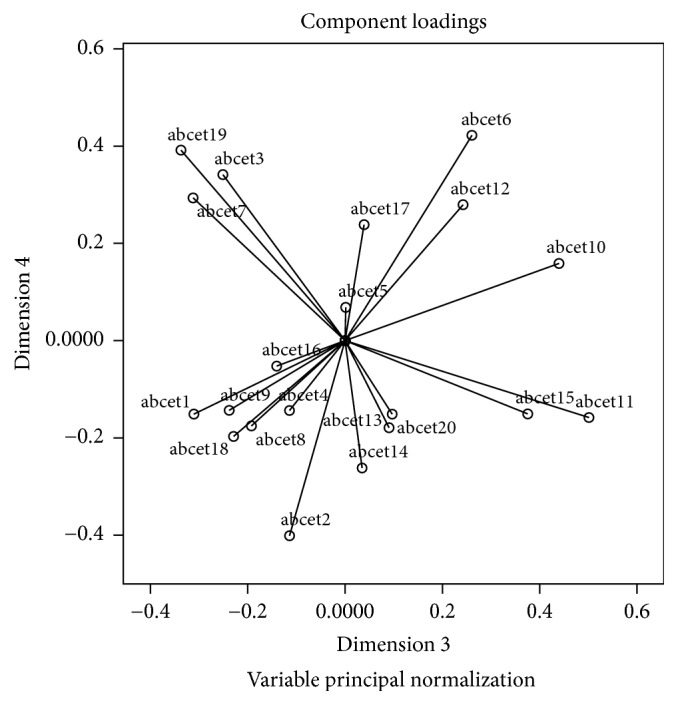
CatPCA biplot of dimensions 3 and 4.

Component loadings are presented in Figures 1–3. The range of component loadings is from −1 to 1 and suggests the correlations between the quantified variables and the principal components. The coordinates of the end point of each vector are the loadings of each item on the two components plotted. Item vectors that are close together in the plot are closely and positively related. Items with vectors that make approximately a 180° angle with each other are closely and negatively related. Variables vectors with a 90° angle are not related [54].

Examination of the biplots suggest that, graphically, the variables do not seem to form distinct or particularly meaningful groups/clusters that might conform to the original 4 estimation domains or that represent some new groupings with clinical meaning.

### 3.2. Item Analysis

Following examination of the biplots, item analysis was used to examine the distribution of responses to each item of the BCET. Examination of the percentage of correct and incorrect responses (Table 2) shows that no item was correctly estimated by >80% of participants. Items (15) (distance/length), (5) (distance/length), and (13) (distance/length) were the most frequently correct with over 75% correct responses. By contrast, items (1) (quantity), (9) (quantity), and (18) (weight) were the most frequently incorrect estimations.

Rotation options are not available in the CatPCA program and given the well-documented difficulty in interpretation of unrotated solutions, the transformed variables were additionally saved and utilised as input variables to permit a rotated solution [53]. The component loadings for the four-dimension analysis are shown in Table 2. Component loadings are arranged in decreasing order within dimensions, loadings greater than 0.40 are in bold, and loadings less than 0.30 are suppressed to aid interpretation except where they denote a possible splitting across factors.  Table 3 shows the 4-factor solution for the BCET and Cronbach's alpha from the original CatPCA analysis and when transformed through CatPCA with Varimax rotation.

Across the subscale assessment dimensions of quantity, weight, time, and distance, individual compatible items did not consistently fall into these established dimensions. Moreover, the factors produced were also not interpretable in any obvious manner (e.g., there was no significant evidence of items perhaps forming clusters where the individual may have been more likely to have had direct procedural experience, such as ironing a shirt, versus those perhaps relying exclusively on semantic information such as estimating how far a horse could pull a farm cart in one hour). Given the difficulty in obtaining a fully interpretable solution in relation to the proposed subscales (quantity, weight, length/distance, and time/duration) as suggested in Figures 1–3 and in Table 3, and low Cronbach's alphas for 3 of the subscales from the original CatPCA analysis, it was deemed prudent to utilise the total score of the BCET in all further examination of the scale in the current sample.

### 3.3. Association of BCET with Clinical and Demographic Factors

#### 3.3.1. Education

Mean total BCET scores rose with educational attainment. This represented a significant effect (*F*(3,197), 6.84, *p* = 0.001) and Scheffe post hoc tests suggested that the effect was largely monotonic, with individuals who had attained a postgraduate education scoring significantly higher on the BCET (BCET mean (SD) for primary school leaver 13.29 (3.17), secondary school leaver 14.36 (2.79), and postgraduate 16.33 (2.49)).

#### 3.3.2. Age, Age at Onset, and Duration with ABI

Higher (better) BCET scores were associated with increasing age of the participant (*r* = .21, *p* = 0.004) and with an older age at onset (*r* = .24, *p* = 0.001), but not with duration with ABI (*r* = −.12, *p* = 0.10).

#### 3.3.3. Cause of ABI

One-way ANOVA suggested that people who had sustained a TBI scored more poorly on the BCET, mean (SD) TBI = 14.04 (2.44), than people who had sustained a stroke, BCET mean SD = 15.44 (3.19), or other causes, mean SD = 15.9, 3.10 (*F*(2,198), 4.73, *p* = 0.01).

#### 3.3.4. Severity of Disability

BCET total score did not significantly differ as a function of the severity of participant's disability as assessed by MPAI-4 *T* score (mean (SD) for mild/moderate disability = 15.09 (2.97); mean (SD) for severe disability = 14.25 (2.81); *t* = 1.58, *p* = 0.11).

#### 3.3.5. Correlations between BCET, Neuropsychological Measures, Disability, and Community Integration

Table 4 shows the relationship between the BCET and the neuropsychological tests with executive aspects (TMT, MSET, Phonemic Fluency), the RBANS, severity of disability (MPAI-4), and community integration (CIQ). BCET was associated most strongly with the Modified Six Elements Test (*r* = .42, *p* = 0.001), phonemic fluency (*r* = .26, *p* = 0.001), TMT B (*r* = −.29, *p* = 0.001), and the TMT difference score (*r* = −.23, *p* = 0.001) but not with the TMT ratio score (*r* = −.07). Additionally, the BCET was also highly correlated with more general indices of neuropsychological functioning including the TMT A (*r* = −.28, *p* = 0.001) and the 6 scales of the RBANS (with values ranging from *r* = .27 for visuospatial processing, to *r* = .43 for immediate memory). Higher BCET scores were also associated with increased community integration (*r* = .25, *p* = 0.001).

#### 3.3.6. Accuracy of Responses

Using the established cut-off for the BCET (scores below 15.6 indicating impairment in estimation abilities), 55.7% of participants had scores in the unimpaired range, with 44.3% scoring in the impaired range. The estimation-impaired group scored significantly lower on the MSET (mean (SD) = 1.93 (1.1) versus 2.76 (1.2); *t* = −4.62, *p* = 0.001), on verbal fluency (mean (SD) = 21.45 (11.34) versus 26.07 (11.85); *t* = −2.68, *p* = 0.006) and semantic fluency (mean (SD) = 13.40 (4.25) versus 15.63 (4.71); *t* = −3.28, *p* = 0.001) and on RBANS indices of immediate memory (mean SD = 67.04 (18.34) versus 79.14 (21.27); *t* = −4.05, *p* = 0.001), visuospatial functioning (mean SD = 79.60 (19.03) versus 88.96 (23.20); *t* = −2.91, *p* = 0.004), language (mean SD = 77.91 (13.57) versus 88.96 (23.20); *t* = −2.95, *p* = 0.004), attention (mean SD = 72.24 (17.56) versus 84.56 (17.41); *t* = −4.57, *p* = 0.001), and delayed memory (mean SD = 66.34 (21.67) versus 79.15 (18.48); *t* = −4.09, *p* = 0.001). The BCET cut-off was not significantly associated with scores on the TMT A mean SD = 69.34 (49.88) versus 57.06 (43.21) and TMT B (mean SD = 204.44 (157.36) versus 184.39 (143.60); *t* = −1.76, *p* = 0.07) or its difference (mean SD = 149.01 (102.11) versus 110.87 (107.04); *t* = −1.19, *p* = 0.23) or ratio score (mean SD = 3.32 (2.33) versus 2.56 (2.20); *t* = −1.29, *p* = 0.19). The cut-off score was not significantly affected by severity of disability (*χ*^2^ = 2.39, *p* = 0.14). Participants with a younger age at onset were more likely to score above the BCET cut-off (mean SD = 29.43 (13.85) versus 37.83 (13.91); *t* = −4.08, *p* = 0.001). A forward conditional logistic regression analysis including the statistically significant univariate results above suggested that BCET cut-off scores were predicted by a younger age at onset of ABI (odds ratio (OR) = 1.04, 95% CI = 1.01–1.06, Wald = 10.49, *p* = 0.001) poorer scores on the MSET (OR = 1.51, 95% CI = 1.11–2.04, Wald = 6.97, *p* = 0.008) and poorer scores on RBANS attention index (OR = 1.03, 95% CI = 1.00–1.05, Wald = 7.19, *p* = 0.007). The model discriminated the unimpaired cognitive estimation group from the unimpaired estimation group with an accuracy of 76.8%.

#### 3.3.7. Prediction of Community Integration

Given the BCET's apparent lack of specificity in assessment in terms of performance based cognitive functioning, the BCET total score was regressed onto participants' community integration scores to examine if the functions assessed by the BCET may explain unique variance in more pragmatic social integration outcomes when entered on the final step in the model. Therefore, a hierarchical multiple regression analysis was undertaken with variables that correlated with community integration as IVs. Thus, severity of disability as assessed by MPAI was entered on step (1), RBANS total score on step (2), MSET on step (3), semantic fluency on step (4), TMT B (as it was the most highly correlated of the TMT measures) on step (5), and BCET on step (6).

Results (Table 5) suggested that the model accounted for 21% of the variance in community integration. Severity of ABI-induced disability, MSET, TMT B, and semantic fluency did not account for significant variance in community integration scores. RBANS total score accounted for 12% of the variance in CIQ scores, and when the BCET was entered on the last step of the model, it accounted for an additional 4% of the variance in community integration.

## 4. Discussion

The current study comprehensively examined the convergent and divergent validity of estimation abilities operationalised through the Biber Cognitive Estimation Test (BCET) in a large sample of individuals with acquired brain injury undergoing postacute rehabilitation. The study examined the latent variable structure of the BCET, reported an item analysis of responses, and addressed whether performance on executive or more general assessments of neuropsychological functioning were more strongly associated with the BCET total score and with its impairment cut-off score. This is also the first study to date to examine the effects of estimation abilities on what is usually considered to be the end point of rehabilitation, namely, the person's level of integration into their community.

### 4.1. Latent Variable Structure

The latent variable structure of the BCET was examined by Categorical Principal Components Analysis, a relatively new algorithmic model which was considered to be more appropriate than more traditional models given that CatPCA can reveal nonlinear relationships by quantifying categorical or nonlinearly related variables to reveal relational structures among the observed variables. Despite examining the BCET with a number of procedures including CatPCA variance accounted for, examining the coordinates for each item on each dimension in relation to the centroid, the use of biplots, and utilising rotation procedures, none of these represented the information in the data sufficiently closely such that it might be reduced in a meaningful way to the smaller number of summary variables (quantity, weight, distance/length, and time/age). Neither were there any obvious theoretically informed justifications for interpretation of the factors, such as components representing a process of estimation that may have relied upon direct procedural experience (e.g., ironing a shirt) versus a process that may have relied on more remote declarative/semantic procedures to effect an estimate (e.g., estimating the length of a giraffe's neck). While it was judged prudent from a psychometric perspective to restrain further analysis of the BCET in the current study to its total score, the prospect that the areas of estimation targeted by the subscales would be isomorphic in clinical practice with rehabilitation patients may not represent a sensible assumption in any case [56]. For example, estimations of magnitude such as “how far,” “how many,” and “how long” have been hypothesised to be bound together through the demands of the motor control system and may not be necessarily independent [57].

### 4.2. Relationship with Clinical Demographics

The current study found a relationship between BCET estimation performance, education, and age, in that more years of formal education, being older, and sustaining their injury at an older age tended to be associated with better estimation performance. While the original development and validation study for the BCET [29] did not find a relationship between BCET performance and education or age, other studies utilising different assessments of estimation performance have found relationships between longer duration of education and better estimation performance in clinical populations [5, 23, 30, 31, 33]. It may be that the better estimation performance observed in older patients and patients who are older when they sustain their brain injury may be due to their ability to compensate for poorer reasoning and self-monitoring through enhanced or retained semantic or factual knowledge. Indeed there is evidence from fluency tasks which suggests that age-related decline in fluid skills of generating novel search strategies, for example, can be offset by age-related improvement in crystallised knowledge [58], suggesting a moderating effect of age on such cognitive processes. In addition, the relationship between education and estimation abilities may result from both of these factors functioning as proxies for cognitive reserve. Cognitive reserve is the amount of cognitive loss that can be sustained in brain injury before reaching a threshold where a demonstrable cognitive impairment becomes evident. Indeed education features in D'Aniello et al. [59] model of estimation and cognitive reserve, and education has also been demonstrated to increase the likelihood of attainment of specific rehabilitation goals related to adjustment and community participation in patients with ABI undergoing rehabilitation [60]. D'Aniello et al. suggest that education may function to preserve crystallised semantic and factual knowledge (general fund of knowledge); this crystallised knowledge has been hypothesised as a cognitive protective factor in older individuals. This may have implications for understanding the results of our data which suggest better estimation performance in older participants or in participants who were older when they sustained their ABI, as well as in individuals who had more years of formal education.

### 4.3. Relationships with Neuropsychological Measures

In terms of the relationship between the BCET and the more established tests of executive function, the BCET showed a stronger relationship with tests involving planning and set shifting (MSET, TMT) than with the verbal fluency component of executive functions. Both the MSET and the TMT could be considered to map onto the response inhibition and set shifting components of Miyake et al.'s [61] model of executive functioning. The highest correlation with executive measures was with the MSET (*r* = .28). Poorer MSET performance suggests a difficulty in alerting the individual to changes that should be taken into account during the task and to faulty reevaluation of goals and monitoring tasks in progress [62], which would have some conceptual overlap with aspects of estimation. However, in the current study, BCET scores were more strongly associated with assessments of cognitive functioning that were not specific tests of executive functioning. The correlations with the RBANS immediate memory index and the attention index were stronger than with the MSET (*r* = 0.42 and *r* = 0.39, resp.). Of the RBANS subscales, immediate memory and attention could be considered more legitimately to require aspects of executive function in their performance, particularly updating semantic working memory. Similarly, in terms of discriminating an impaired estimation group from an unimpaired group, patients whose BCET scores placed them in the impaired category were significantly more likely to have a younger age at onset of their brain injury, lower MSET scores, and poorer RBANS attention index scores.

### 4.4. Prediction of Rehabilitation Outcome

The distal goal of postacute rehabilitation is to integrate the person into their community. While the assessed model accounted for a relatively modest proportion of the variance in community integration (21%), some important findings were uncovered. Namely, that the RBANS total score accounted for the majority of the variance in community integration, with severity of ABI-induced disability, MSET, TMT B, and semantic fluency not making any significant contribution to the model. The BCET however did account for unique variance in community integration after it was entered on the last step of the model accounting for an additional 4% of the variance. While modest, it nonetheless suggests that the BCET may be assessing something distinctive and of potential value in this area. Further work needs to be undertaken to develop the assessment protocol for cognitive estimation including various forms of presentation that may involve visual cueing as an attention orientation strategy. Similarly, goal management interventions [63] may prove helpful in moderating the effects of estimation challenges as part of a real-world goal management training approach. Such an approach may target executive attention with the intention of enabling the person with an ABI to halt on-going behaviour and cognition periodically, such that the person can monitor and adjust their behaviour in relation to immediate real-world estimation goals in their community.

### 4.5. Limitations

The study has some limitations. Firstly neuroimaging data was not available on participants and therefore no detailed formal information can be provided from a structural-functional perspective on anterior versus posterior injuries or in terms of issues of laterality and their possible effects on BCET performance. However, since brain injury patients with moderate to severe injuries typically have large lesions affecting fairly large tracts of frontal and temporoparietal cortex, it is also possible that multiple functional systems may be affected which would limit the ability of the BCET to localise function in such samples in any case. Secondly, variability in the ages at which participants sustained their brain injury, while reflecting the nature of routine referrals to ABI postacute rehabilitation, may have influenced the results given issues of brain plasticity in more established versus more recent injury. Thirdly the sample comprised people who had sustained brain injuries of varying causes; thus, standard severity measures which might be utilised in TBI cases were not sensible to be used (e.g., GCS, duration of PTA, and LOC) and that criteria for severity in CVA such as Rankin criteria are not routinely utilised in the Republic of Ireland. Therefore, a valid and reliable measure of disability arising from acquired brain injury (MPAI-4) was used in order to provide a sympathetic measure that would have relevance to all types of ABI. The presence of clinical severity information from the time the person sustained their injury in addition to specific and contemporary lesion location information would have been a helpful adjunct to the study.

### 4.6. Conclusions

This is the first study to examine the effects of estimation abilities on community integration in a rehabilitation sample of people with acquired brain injury. The study has also provided detailed information on estimation assessment in this patient group. While the latent structure of the BCET in the current study did not readily map onto the four subscale indices of quantity, weight, distance/length, and time/age and to describe the BCET solely as a test of executive functioning in the current rehabilitation sample would not be sensible, the measure has some merit in terms of its association with pragmatic outcomes and may have a continued role in assessment protocols in postacute rehabilitation.

## Figures and Tables

**Table 1:** Means and standard deviations of the assessments used in the study.

Test	Mean (SD)	Range
Biber Cognitive Estimation Test (BCET)	14.67 (2.89)	9–20
Trail-Making Test		
TMT-A	64.50 (47.37)	18–247
TMT-B	190.03 (154.98)	41–300
TMT-B/A	2.94 (2.27)	0.42–21.74
TMT-B − A	125.53 (104.04)	14–287
*Verbal fluency*		
Phonemic fluency	23.38 (11.85)	0–55
Semantic fluency	14.29 (4.59)	0–30
MSET	2.21 (1.25)	0–4
RBANS		
Immediate memory	72.24 (20.72)	14–120
Visuospatial processing	83.74 (21.31)	50–121
Language	80.51 (14.94)	9–120
Attention	77.39 (18.64)	40–115
Delayed memory	71.78 (21.39)	21–122
Total scale score	71.52 (17.05)	40–115
Community integration scale	14.85 (4.72)	2–24
MPAI-*T* score	39.03 (12.52)	4–65

MSET = Modified Six Elements Test; RBANS = Repeatable Battery for Assessment of Neuropsychological Status; MPAI = Mayo Portland Adaptability Index.

**Table 2:** Item analysis and component analysis of the BCET.

Item	% correct	% incorrect	1	2	3	4
(10) How many crisps are there in a small one-ounce bag?	72.6	27.4	**.671**			
(11) How long would it take an adult to handwrite a one-page letter?	62.2	37.8	**.649**			
(15) How long does it take to iron a shirt?	80.1	19.9	**.618**			
(12) What is the age of the oldest living person in the USA?	65.2	34.8	**.546**			
(6) How long does it take a builder to build an average sized house?	72.1	27.9	**.518**			
(17) How many slices of bread are there in a one-pound loaf?	70.1	29.9	**.417**			
(20) How long does it take for fresh milk to go sour in the refrigerator?	59.2	40.8	**.411**			
(2) How much does a (house) telephone weigh?	74.6	25.4		**.734**		
(14) How much does a folding chair weigh?	73.1	26.9		**.696**		
(18) How much does a pair of men's shoes weigh?	56.2	43.8		**.692**		
(13) How long is a tablespoon?	75.6	24.4	.416	**.462**		
(5) How high off a trampoline can a person jump?	79.1	20.9	.376	**.417**	.337	
(16) How long is a giraffe's neck?	75.1	24.9		**.400**	.378	
(19) How much does the heaviest man in the United States weigh?	66.7	33.3			**.713**	
(4) What is the distance an adult can walk in an afternoon?	69.7	30.3			**.656**	
(7) How much do a dozen, medium-sized apples weigh?	57.7	42.3			**.513**	
(3) How many sticks of spaghetti are there in a one-pound package?	63.2	36.8				**.702**
(9) How many brushings can someone get from a large tube of toothpaste?	53.7	46.3				**.696**
(1) How many seeds are there in a watermelon?	51.2	48.8				**.500**
(8) How far could a horse pull a farm cart in one hour?	73.6	26.4				**.401**

**Table 3:** Component solutions for BCET and Cronbach's alpha from original CatPCA analysis and when transformed through CatPCA and rotated via Varimax rotation.

	CatPCA			Transformed through CatPCA and rotated		
	Eigenvalue	VAF (% variance)	Alpha	Eigenvalue	% variance	Alpha
Factor 1	5.75	28.70	0.87	2.75	13.77	0.77
2	1.38	6.89	0.29	2.51	12.54	0.72
3	1.25	6.48	0.24	2.16	10.84	0.58
4	1.17	5.84	0.15	2.16	10.83	0.57

Total	9.581	47.91	0.94			0.86

**Table 4:** Pearson correlations between BCET and neuropsychological measures.

	1	2	3	4	5	6	7	8	9	10	11	12	13	14	15
(1) B-CET	1														
(2) MSET	**.284** ^*∗∗*^	1													
(3) TMT (A)	−**.253**^*∗∗*^	−**.405**^*∗∗*^	1												
(4) TMT (B)	−**.242**^*∗∗*^	−**.344**^*∗∗*^	**.518** ^*∗∗*^	1											
(5) TMT (B/A)	−**.212**^*∗∗*^	−.101	−**.301**^*∗∗*^	**.660** ^*∗*^	1										
(6) TMT (B − A)	**.270** ^*∗∗*^	−**.263**^*∗∗*^	**.197** ^*∗*^	**.857** ^*∗∗*^	**.804** ^*∗∗*^	1									
(7) Phonemic Fluency	**.260** ^*∗∗*^	**.409** ^*∗∗*^	−**.420**^*∗∗*^	−**.384**^*∗∗*^	−.008	−**.211**^*∗∗*^	1								
(8) Semantic Fluency	**.225** ^*∗∗*^	**.275** ^*∗∗*^	−**.336**^*∗∗*^	−**.391**^*∗∗*^	−.139	−.322	**.184** ^*∗*^	1							
(9) RBANS IM	**.426** ^*∗∗*^	**.365** ^*∗∗*^	−**.300**^*∗∗*^	−**.317**^*∗∗*^	−.017	−**.148**^*∗*^	**.465** ^*∗∗*^	**.169** ^*∗*^	1						
(10) RBANS VS	**.276** ^*∗∗*^	**.289** ^*∗∗*^	−**.339**^*∗∗*^	−**.294**^*∗∗*^	−.081	−**.157**^*∗*^	**.283** ^*∗∗*^	**.245** ^*∗∗*^	**.416** ^*∗∗*^	1					
(11) RBANS Lang	**.286** ^*∗∗*^	**.177** ^*∗*^	−**.246**^*∗∗*^	−**.192**^*∗*^	−.112	−**.217**^*∗∗*^	**.406** ^*∗∗*^	**.246** ^*∗∗*^	**.516** ^*∗∗*^	**.287** ^*∗∗*^	1				
(12) RBANS Att	**.395** ^*∗∗*^	**.283** ^*∗∗*^	−**.395**^*∗∗*^	−**.384**^*∗∗*^	−.110	−**.309**^*∗∗*^	**.463** ^*∗∗*^	**.188** ^*∗*^	**.512** ^*∗∗*^	**.340** ^*∗∗*^	**.400** ^*∗∗*^	1			
(13) RBANS DM	**.335** ^*∗∗*^	**.276** ^*∗∗*^	−**.302**^*∗∗*^	−**.239**^*∗∗*^	−.118	−**.176**^*∗*^	**.364** ^*∗∗*^	**.210** ^*∗∗*^	**.633** ^*∗∗*^	**.466** ^*∗∗*^	**.376** ^*∗∗*^	**.467** ^*∗∗*^	1		
(14) RBANS total	**.481** ^*∗∗*^	**.367** ^*∗∗*^	−**.386**^*∗∗*^	−**.361**^*∗∗*^	−.133	−**.301**^*∗∗*^	**.532** ^*∗∗*^	**.268** ^*∗∗*^	**.818** ^*∗∗*^	**.711** ^*∗∗*^	**.590** ^*∗∗*^	**.705** ^*∗∗*^	**.814** ^*∗∗*^	1	
(15) CIQ total	**.249** ^*∗∗*^	**.209** ^*∗∗*^	−**.144**^*∗*^	−**.152**^*∗*^	−.055	.119	.104	**.226** ^*∗∗*^	**.156** ^*∗*^	.129	.121	**.177** ^*∗*^	**.294** ^*∗∗*^	**.266** ^*∗∗*^	1
(16) MPAI *T* score	−.026	.086	.122	.103	−.101	.017	.105	.090	−.076	.011	**.207** ^*∗∗*^	−.099	.001	−.020	−**.218**^*∗∗*^

^*∗*^ *p* < 0.05, ^*∗∗*^*p* < 0.01; BCET = Biber Cognitive Estimation Test; MSET = Modified Six Elements Test; TMT = Trail-Making Test; RBANS = Repeatable Battery for Assessment of Neuropsychological Status; IM = immediate memory Index; VS = visuospatial/constructional index; Lang = language index; Att = attention index; DM = delayed memory; CIQ = Community Integration Questionnaire; MPAI = Mayo Portland Adaptability Index.

**Table 5:** Hierarchical multiple regression of severity of disability, RBANS total score, MSET, TMT-B, semantic fluency, and BCET onto community integration.

Step	Δ*R*2	*β*	*t*	*p*	95% CI
(1) Severity of disability (MPAI-4)	.028	−.207	1.91	0.06	−.14 to −.04
(2) RBANS total score	.136	.245	2.29	0.02	0.12 to .16
(3) MSET	.133	.050	0.41	0.68	−0.65 to 0.98
(4) Semantic fluency	.123	−.041	0.03	0.97	−0.23 to 0.22
(5) TMT-B	.125	.104	0.92	0.35	−1.55 to −4.26
(6) B-CET	.160	.246	2.16	0.03	0.30 to 0.69

MPAI-4 = Mayo Portland Adaptability Index; RBANS = Repeatable Battery for Assessment of Neuropsychological Status; MSET = Modified Six Elements Test; TMT-B = Trail-Making Test form B; BCET = Biber Cognitive Estimation Test.
